# Functional identification of potential non-canonical N-glycosylation sites within Ca_v_3.2 T-type calcium channels

**DOI:** 10.1186/s13041-020-00697-z

**Published:** 2020-11-11

**Authors:** Vendula Ficelova, Ivana A. Souza, Leos Cmarko, Maria A. Gandini, Robin N. Stringer, Gerald W. Zamponi, Norbert Weiss

**Affiliations:** 1 Institute of Biology and Medical Genetics, First Faculty of Medicine, Charles University, Prague, Czech Republic; 2 Institute of Organic Chemistry and Biochemistry, Czech Academy of Sciences, Prague, Czech Republic; 3 Department of Physiology and Pharmacology, Cumming School of Medicine, University of Calgary, Calgary, Canada; 4 Third Faculty of Medicine, Charles University, Prague, Czech Republic

**Keywords:** Asparagine-linked glycosylation, N-glycosylation, Non-canonical glycosylation, Calcium channel, T-type channel, ca_v_3.2 Channel, Trafficking

## Abstract

Low-voltage-activated T-type calcium channels are important contributors to nervous system function. Post-translational modification of these channels has emerged as an important mechanism to control channel activity. Previous studies have documented the importance of asparagine (N)-linked glycosylation and identified several asparagine residues within the canonical consensus sequence N-*X*-S/T that is essential for the expression and function of Ca_v_3.2 channels. Here, we explored the functional role of non-canonical N-glycosylation motifs in the conformation N-*X*-C based on site directed mutagenesis. Using a combination of electrophysiological recordings and surface biotinylation assays, we show that asparagines N345 and N1780 located in the motifs NVC and NPC, respectively, are essential for the expression of the human Ca_v_3.2 channel in the plasma membrane. Therefore, these newly identified asparagine residues within non-canonical motifs add to those previously reported in canonical sites and suggest that N-glycosylation of Ca_v_3.2 may also occur at non-canonical motifs to control expression of the channel in the plasma membrane. It is also the first study to report the functional importance of non-canonical N-glycosylation motifs in an ion channel.

Low-voltage-activated T-type calcium channels are widely expressed throughout the nervous system where they generate low-threshold calcium spikes that contribute to neuronal electrical excitability [1]. Over recent years, post-translational modification of the channel protein including phosphorylation [2–4], ubiquitination [5], and glycosylation [6] has emerged as an important level of control over the expression and function of the channel in the plasma membrane, and alteration of these regulations is known to contribute to the development of several neurological disorders. Therefore, the identification of channel loci undergoing post-translational modification is essential not only to enhance our fundamental understanding of the channel, but also to gain insights into how alteration of these regulations may compromise channel function in pathological conditions.

We and others have previously documented the importance of asparagine (N)-linked glycosylation in the expression of the Ca_v_3.2 T-type channels and identified several asparagines essential for the expression of the channel in the plasma membrane [7, 8]. These asparagine residues are located within the sequence N-*X*-S/T commonly referred to as the canonical N-glycosylation motif where the asparagine is located at the N-terminal to any amino acid (except proline) followed by either a serine (S) or threonine (T). However, while N-glycosylation at N-*X*-S/T motifs is an established dogma, there is evidence for the occurrence of N-glycosylation at non-canonical motifs falling into the conformation N-*X*-C (cysteine) [9]. In the present study, we aimed to further explore the glycosylation loci of Ca_v_3.2 channels and assess whether asparagines located within such non-canonical motifs contribute to the expression of the channel. The human Ca_v_3.2 channel contains four potential non-canonical motifs defined by asparagines N258, N335 and N345 located within the first pore-forming loop (P-loop), and asparagine N1780 within the forth P-loop of the channel (Fig. 1a) and in-silico analysis using NetNGlyc 1.0 server (https://www.cbs.dtu.dk/services/NetNGlyc/) predicted that these sites could be potentially glycosylated (Fig. 1b). To assess the functional importance of these residues in the expression of Ca_v_3.2 channels, we used site directed mutagenesis to disrupt these motifs. We replaced asparagine residues with glutamine (Q) and such recombinant mutated channels were expressed in tsA-201 cells for functional characterization by patch clamp electrophysiology. Representative current traces for cells expressing the wild-type (WT) channel and the various mutated variants (N258Q, N335Q, N345Q, and N1780Q) are shown in Fig. 1c. While all channel variants produced a characteristic low-threshold voltage-activated T-type current, currents recorded from cells expressing the N345Q and N1780Q variants were strongly reduced compared to cells expressing the WT channel (Fig. 1d). The maximal whole cell slope conductance was reduced by 50% (*p* = 0.0050) in N345Q (480 ± 68 pS/pF, n = 17) and by 56% (*p* = 0.0021) in N1780Q-expressing cells (423 ± 90 pS/pF, n = 11) compared to cells expressing the WT channel (970 ± 110 pS/pF, n = 27). This decrease of the maximal conductance was associated with a mild but significant shift of the voltage-dependence of activation without any additional alteration of the voltage-dependence of inactivation and recovery from inactivation (Additional file 1: Figure S1). Furthermore, mutations at asparagines N345 and N1780 did not alter the ability of nickel to block T-type currents (Additional file 1: Figure S2). Next, we aimed to determine whether the impaired T-type conductance in cells expressing the N345Q and N1780Q channels was caused by an alteration of the channel activity or due to reduced expression of the channels in the plasma membrane. To do so, we used cell surface biotinylation followed by immunodetection of the channels. Representative immunoblots of total and surface biotinylated channels are shown in Fig. 1f and g, respectively. No immunoreactivity was detected in non-biotinylated cells expressing WT Ca_v_3.2 channels demonstrating the absence of contamination from other cellular fractions and also the absence of non-specific interaction of the channel with NeutrAvidin beads. While the total channel expression was statistically similar across all channel variants (Fig. 1f), the expression of the N345Q and N1780Q channels in the plasma membrane was reduced by 50% (*p* = 0.0031) and 56% (*p* = 0.0301) respectively, a decrease that is closely correlated with the reduction of the whole cell conductance.

**Fig. 1:**
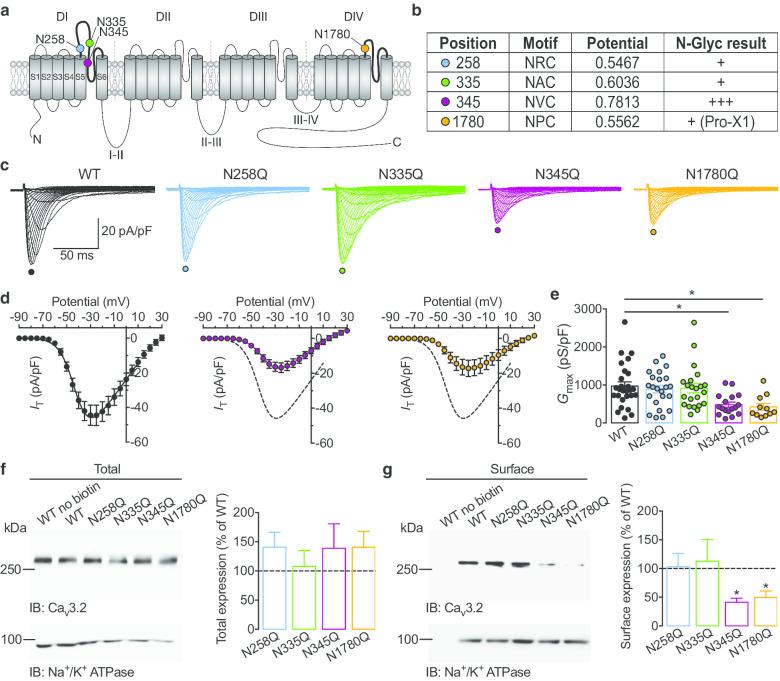
Functional and biochemical analysis of glycosylation-deficient Ca_v_3.2 channels. **a** Schematic representation of the membrane topology of Ca_v_3.2 channel depicting the localization of asparagine residues within potential non-canonical glycosylation motifs. **b** Result of the in-silico N-glycosylation prediction. Position of the key asparagine residues (N) within the potential glycosylation motif (N*X*C) is indicated, along with the quantitative (threshold 0.5) and qualitative score. *Pro-X1* indicates the presence of a proline residue in position + 1 of the asparagine residue which is known to limit the likelihood for glycosylation. **c** Representative T-type current traces recorded from tsA-201 cells expressing wild-type (WT) Ca_v_3.2 channels (black traces), and N258Q (blue traces), N335Q (green traces), N345Q (purple traces), N1780Q (orange traces) mutated channels in response to 150 ms depolarizing steps to values ranging from − 90 to + 30 mV from a holding potential of − 100 mV. **d** Corresponding mean peak current density–voltage (*I*/*V*) relationship. The *I*/*V* curve of the WT channel is reported in dotted line for comparison. **e** Corresponding mean normalized maximal macroscopic conductance (*G*_max_) values obtained from the fit of the *I*/*V* curves with a modified Boltzmann equation. **f** Representative immunoblot of Ca_v_3.2 channel variants from total cell lysate (total expression, left panel) and corresponding mean total expression as percent of WT channels. The expression of the channels was normalized to Na^+^/K^+^ ATPase. **g** Legend same as in (**f**) but for surface biotinylated fractions (surface expression). The biochemical analysis was repeated five times from independent transfections with similar results

Glycosylation of T-type channels has emerged as an important post-translational modification to control the expression and functioning of the channel in the plasma membrane, and it was suggested that alteration of the glycoproteome of Ca_v_3.2 may contribute to the development of peripheral pain associated with diabetes [8, 10]. While several studies have previously reported the importance of canonical glycosylation sites in the expression and function of Ca_v_3.2 channels [7, 8, 11, 12], the potential role for non-canonical motifs has never been explored. Here, we identified two asparagine residues, N345 and N1780 located within non-consensus glycosylation motifs in the conformation N-*X*-C that contribute to the expression of the channel in the plasma membrane. Although the exact underlying mechanisms by which these two asparagines influence the expression of the channel at the cell surface was not explored in this study, it is likely that they either enhance the trafficking of the channel to the cell surface, or stabilize the channel protein in the plasma membrane by slowing down its internalization as it was previously reported for other glycosylation loci [11]. Because of the large molecular weight of the full-length channel and the existence of several canonical glycosylation sites, it is challenging to demonstrate by Western blot analysis that mutagenesis of the non-canonical sites leads to small molecular weight shifts that are consistent with fewer sugar groups. We can therefore not exclude the possibility that alteration of Ca_v_3.2 expression upon mutagenesis of asparagines N345 and N1780 may have resulted from an alteration of the channel itself rather than from disruption of its glycosylation. However, this mutagenesis approach is commonly used to functionally assess the functional importance of glycosylation motifs, and glutamine as a replacement of asparagine was chosen because of its similarity, which is therefore expected to preserve the local charge distribution and secondary structure of the channel. Moreover, we cannot totally exclude that mutagenesis of asparagines N345 and N1780 may have interfered with the ubiquitination of the channel, although this process occurs at a different locus (III-IV linker) and therefore is not expected to be directly impacted by the mutations [5]. Our observation that the total expression of mutated channels remained unaltered would also argue against an effect on the ubiquitination pathway.

Altogether, this study supports the notion that Ca_v_3.2 channels may undergo N-glycosylation at non-canonical motifs and identified two sites defined by asparagines N345 and N1780 important for expression of the channel at the cell surface. To our knowledge, this study is the first to document the functional role of non-canonical N-glycosylation motifs within an ion channel.

## Supplementary information

Additional file 1. **Figure S1** Electrophysiological properties of Ca_v_3.2 channel variants. **a** Mean normalized voltage-dependence of activation for wild-type (WT) Ca_v_3.2 channels (black circles), and N258Q (blue circles), N335Q (green circles), N345Q (purple circles), N1780Q (orange circles). **b** Corresponding mean half-activation potential values obtained from the fit of the activation curves with a modified Boltzmann equation. **c-d** Legend same as for (a-b) but for the voltage-dependence of steady state inactivation. **e** Mean normalized recovery from inactivation kinetics. **f** Corresponding mean time constant values of recovery from inactivation obtained from the fit of the recovery curves with a single-exponential function. **Figure S2** Effect of nickel on Ca_v_3.2 channel variants. **a** Representative T-type current traces recorded from tsA-201 cells expressing wild-type (WT, black traces), N345Q (purple traces) and N1780Q (orange traces) Ca_v_3.2 variants recorded in response to 150 ms depolarizing steps to -20 mV from a holding potential of -100 mV before (Ctrl) and after application of 50 μM nickel (Ni^+^). **b** Corresponding mean peak current inhibition.

## Data Availability

All data generated or analyzed during this study are included in this published article and its supplementary information files.
