# Supplementary material for: Functional identification of potential non-canonical N-glycosylation sites within Cav3.2 T-type calcium channels
Source: Mol Brain. 2020 Nov 11;13:149. doi: 10.1186/s13041-020-00697-z (PMC7659234; doi:10.1186/s13041-020-00697-z)
Supplement: Supplementary file 1 — Additional file 1. Figure S1 Electrophysiological properties of Cav3.2 channel variants. a Mean normalized voltage-dependence of activation for wild-type (WT) Cav3.2 channels (black circles), and N258Q (blue circles), N335Q (green circles), N345Q (purple circles), N1780Q (orange circles). b Corresponding mean half-activation potential values obtained from the fit of the activation curves with a modified Boltzmann equation. c-d Legend same as for (a-b) but for the voltage-dependence of steady state inactivation. e Mean normalized recovery from inactivation kinetics. f Corresponding mean time constant values of recovery from inactivation obtained from the fit of the recovery curves with a single-exponential function. Figure S2 Effect of nickel on Cav3.2 channel variants. a Representative T-type current traces recorded from tsA-201 cells expressing wild-type (WT, black traces), N345Q (purple traces) and N1780Q (orange traces) Cav3.2 variants recorded in response to 150 ms depolarizing steps to -20 mV from a holding potential of -100 mV before (Ctrl) and after application of 50 μM nickel (Ni+). b Corresponding mean peak current inhibition. [file 13041_2020_697_MOESM1_ESM.docx]

**Additional information**

**Additional methods**

**Plasmid cDNA constructs**

The human wild-type Ca_v_3.2 (GenBank NM_021098.2) in pcDNA3.1 was used as a template to disrupt the four potential non-canonical N-glycosylation sites (N-*X*-C) by substituting the asparagine residues N258, N335, N345, and N1780 with glutamine (Q) using QuikChange site-directed mutagenesis kit (Agilent Technologies). After mutagenesis, constructs were verified by sequencing of the cloned region.

***Cell culture and heterologous expression***

Human embryonic kidney tsA-201 cells were grown in DMEM medium supplemented with 10% fetal bovine serum and 1% penicillin/streptomycin (all media purchased from Invitrogen) and maintained under standard conditions at 37^o^C in a humidified atmosphere containing 5% CO_2_. Heterologous expression of recombinant Ca_v_3.2 channel variants was performed by transfecting cells with 5 μg plasmid cDNAs using the calcium/phosphate method.

***Patch-clamp electrophysiology***

Patch clamp recording of T-type current in tsA-201 cells expressing Ca_v_3.2 channel variants was performed in the whole-cell configuration at room temperature (22-24^o^C). The bath solution contained (in millimolar): 5 BaCl2, 5 KCl, 1 MgCl2, 128 NaCl, 10 TEA-Cl, 10 D-glucose, 10 4-(2-hydroxyethyl)-1-piperazineethanesulfonic acid (HEPES) (pH 7.2 with NaOH). Patch pipettes were filled with a solution containing (in millimolar): 110 CsCl, 3 Mg-ATP, 0.5 Na-GTP, 2.5 MgCl2, 5 D-glucose, 10 EGTA, and 10 HEPES (pH 7.4 with CsOH), and had a resistance of 2–4 MΩ. Recordings were performed using an Axopatch 200B amplifier (Axon Instruments) and acquisition and analysis were performed using pClamp 10 and Clampfit 10 software, respectively (Axon Instruments). The linear leak component of the current was corrected online and current traces were digitized at 10 kHz and filtered at 2 kHz. The voltage dependence of activation of Ca_v_3.2 channels was determined by measuring the peak T-type current amplitude in response to 150 ms depolarizing steps to various potentials applied every 10 s from a holding membrane potential of -100 mV. The current-voltage relationship (I/V) curve was fitted with the following modified Boltzmann equation (1):

$$\left( 1 \right) I\left( V \right)= Gmax \frac{(V-Vrev)}{1+ \exp\frac{(V0.5-V)}{k}}$$

with *I*(*V*) being the peak current amplitude at the command potential *V*, *G*max the maximum conductance, *V*rev the reversal potential, *V*_0.5_ the half-activation potential, and *k* the slope factor. The voltage dependence of the whole-cell Ba^2+^ conductance was calculated using the following modified Boltzmann equation (2):

$$\left( 2 \right) G\left( V \right)= \frac{Gmax}{1+ \exp\frac{(V0.5-V)}{k}}$$

with *G*(*V*) being the Ba^2+^ conductance at the command potential *V*.

The voltage dependence of the steady-state inactivation of Ca_v_3.2 channels was determined by measuring the peak T-type current amplitude in response to a 150 ms depolarizing step to -20 mV applied after a 5 s-long conditioning prepulse ranging from -120 mV to -30 mV. The current amplitude obtained during each test pulse was normalized to the maximal current amplitude and plotted as a function of the prepulse potential. The voltage dependence of the steady-state inactivation was fitted with the following two-state Boltzmann function (3):

$$\left( 3 \right) I\left( V \right)= \frac{Imax}{1+ \exp\frac{(V-V0.5)}{k}}$$

with *I*_max_ corresponding to the maximal peak current amplitude and *V*_0.5_ to the half-inactivation voltage.

The recovery from inactivation was assessed using a double-pulse protocol from a holding potential of -100 mV. The cell membrane was depolarized for 2 s at 0 mV (inactivating prepulse) to ensure complete inactivation of the channel, and then to -20 mV for 150 ms (test pulse) after an increasing time period (interpulse) ranging between 0.1 ms and 7 s at -100 mV. The peak current from the test pulse was plotted as a ratio of the maximum prepulse current versus interpulse interval. The data were fitted with the following single-exponential function (4):

$$\left( 4 \right) \frac{I}{Imax}=A \times(1- exp\frac{-t}{\tau})$$

where τ is the time constant for channel recovery from inactivation.

***Cell surface biotinylation***

Surface proteins from transfected cells with 3 μg of plasmids encoding Ca_v_3.2 WT or N258Q, N335Q, N345Q and N1780Q variants were biotinylated for 1 h on ice with 1 mg/mL of EZ-Link Sulfo-NHS-SS-Biotin (Thermo Scientific). The reaction was quenched with 100 mM glycine for 15 min, and cells were lysed in modified RIPA buffer containing in mM: 50 Tris, 150 NaCl, 5 EDTA, 1% Triton X-100, 1% NP-40, 0.2% SDS, pH 7.4 for 30 min. Two mg of lysates were incubated with 100 μL of Neutravidin beads (Thermo Scientific) for 90 min at 4 °C. Beads were washed and proteins eluted with 2× Laemmli sample buffer. Biotinylated proteins and lysates were resolved by SDS-PAGE and analyzed by western blot using the antibodies anti-Ca_v_3.2 (dilution 1:1000, Novus Biologicals NBP1-22444) and anti-Na^+^/K^+^ ATPase (dilution 1:5000, Abcam AB 7671).

***Statistical analysis***

Data values are presented as mean ± S.E.M for *n* measurements. Statistical analysis was performed using GraphPad Prism 7 and statistical significance was determine using a one-way ANOVA test. Datasets were considered significantly different for p ≤ 0.05 *.

**Additional figures**

**
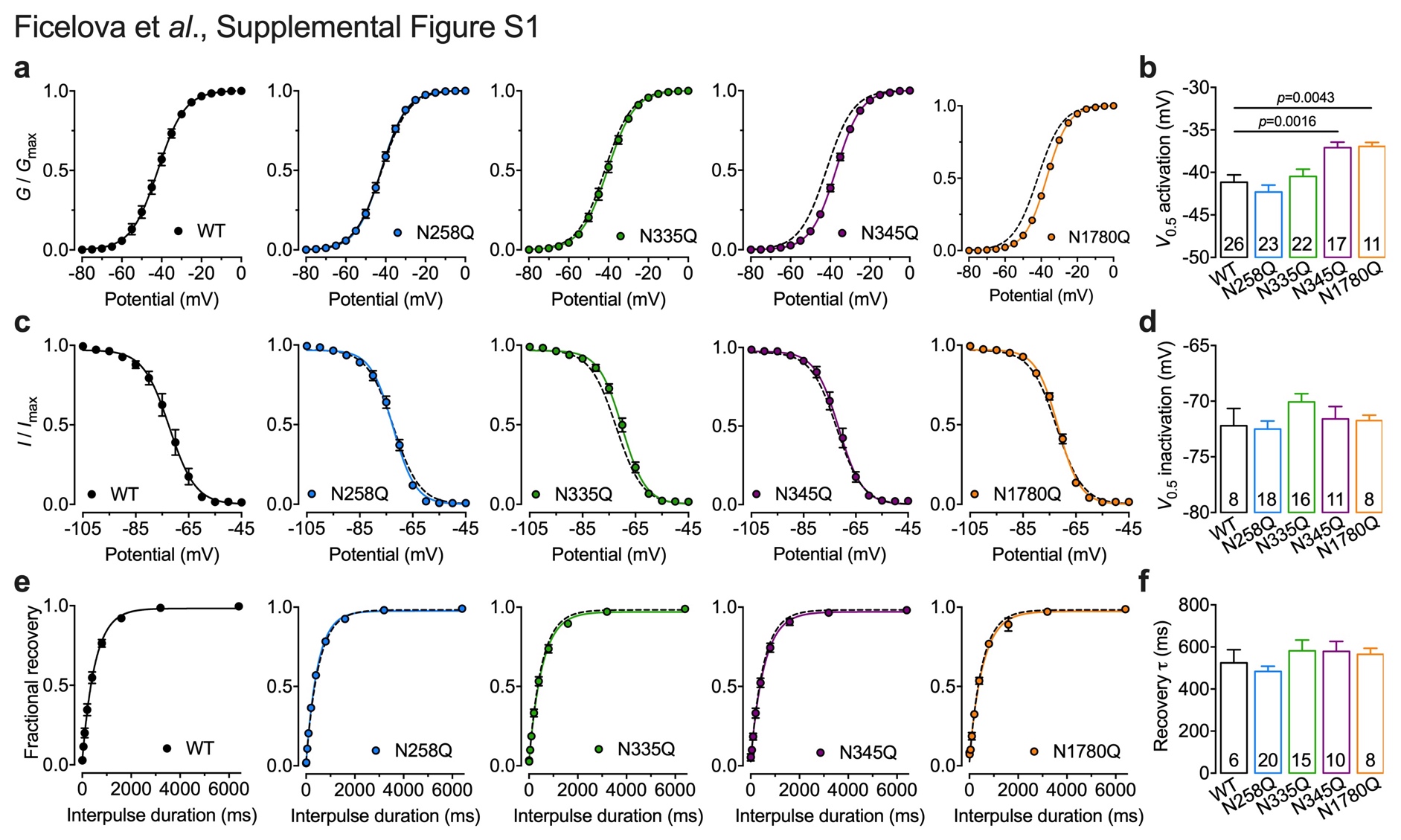
Fig. S1** Electrophysiological properties of Ca_v_3.2 channel variants. **a** Mean normalized voltage-dependence of activation for wild-type (WT) Ca_v_3.2 channels (black circles), and N258Q (blue circles), N335Q (green circles), N345Q (purple circles), N1780Q (orange circles). **b** Corresponding mean half-activation potential values obtained from the fit of the activation curves with a modified Boltzmann equation. **c-d** Legend same as for (a-b) but for the voltage-dependence of steady state inactivation. **e** Mean normalized recovery from inactivation kinetics. **f** Corresponding mean time constant values of recovery from inactivation obtained from the fit of the recovery curves with a single-exponential function.


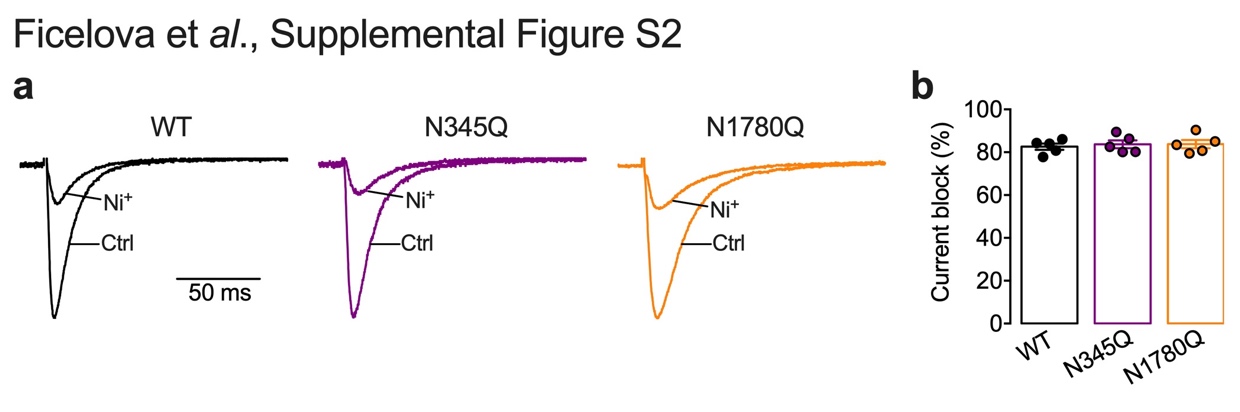


**Fig. S2** Effect of nickel on Ca_v_3.2 channel variants. **a** Representative T-type current traces recorded from tsA-201 cells expressing wild-type (WT, black traces), N345Q (purple traces) and N1780Q (orange traces) Ca_v_3.2 variants recorded in response to 150 ms depolarizing steps to -20 mV from a holding potential of -100 mV before (Ctrl) and after application of 50 μM nickel (Ni^+^). **b** Corresponding mean peak current inhibition.

**Raw data**

***Figure 1F and G***

**
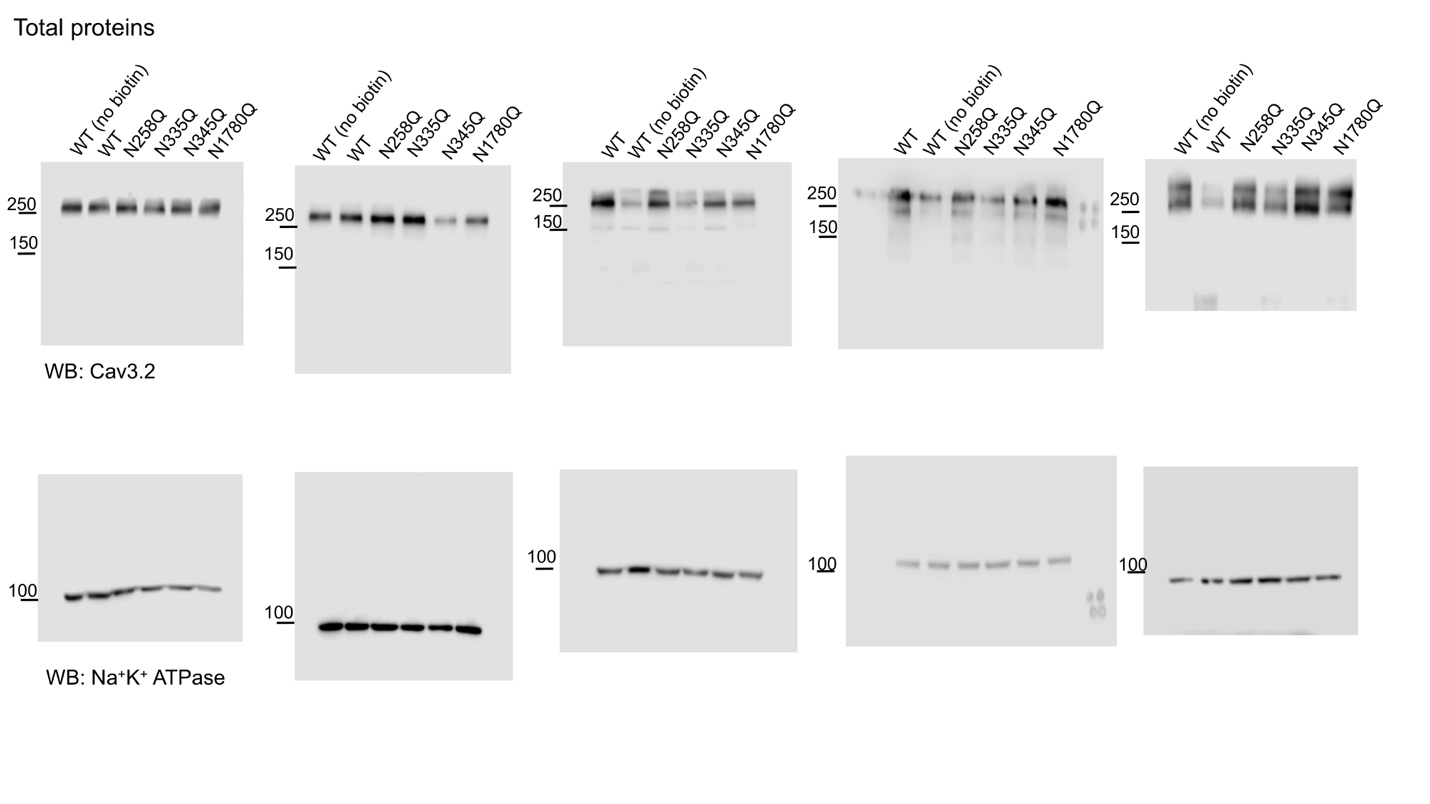
**

**
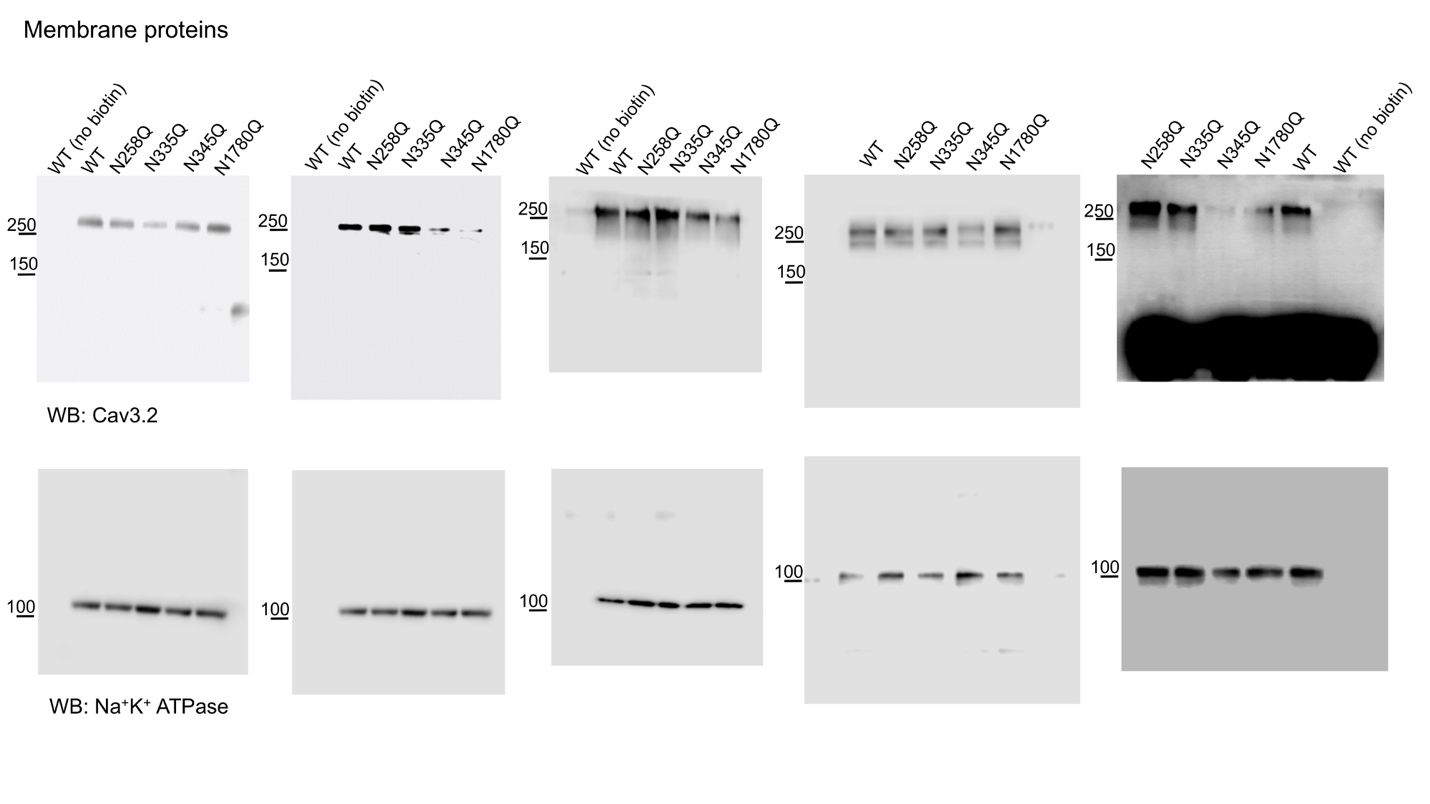
**

**Quantification - Total**

|  | Cav3.2 |  | NaK ATPase |  | Ratio |
| --- | --- | --- | --- | --- | --- |
|  |  |  |  |  |  |
| **Blot 1** |  |  |  |  |  |
|  |  |  |  |  |  |
| WT | 20147.3 |  | 13344.752 |  | 1.877641 |
| N258Q | 22617.91 |  | 11323.217 |  | 1.997481 |
| N334Q | 18362.42 |  | 7694.004 |  | 2.386588 |
| N345Q | 22514.08 |  | 7032.64 |  | 3.201369 |
| N1780Q | 22405.61 |  | 7112.933 |  | 3.149982 |
|  |  |  |  |  |  |
| **Blot 2** |  |  |  |  |  |
|  |  |  |  |  |  |
| WT | 12334.05 |  | 15026.89 |  | 0.820799 |
| N258Q | 18792.64 |  | 14133.803 |  | 1.329624 |
| N334Q | 18491.57 |  | 11654.146 |  | 1.586695 |
| N345Q | 4388.196 |  | 9709.317 |  | 0.451957 |
| N1780Q | 9644.359 |  | 14558.874 |  | 0.662439 |
|  |  |  |  |  |  |
| **Blot 3** |  |  |  |  |  |
|  |  |  |  |  |  |
| WT | 27508.14 |  | 15652.615 |  | 1.757415 |
| N258Q | 24319.05 |  | 10194.288 |  | 2.385556 |
| N334Q | 8056.288 |  | 8067.853 |  | 0.998567 |
| N345Q | 18140.69 |  | 9131.924 |  | 1.986513 |
| N1780Q | 14677.08 |  | 6990.439 |  | 2.099593 |
|  |  |  |  |  |  |
| **Blot 4** |  |  |  |  |  |
|  |  |  |  |  |  |
| WT | 16485.71 |  | 9533.64 |  | 1.729215 |
| N258Q | 12463.16 |  | 9664.175 |  | 1.289625 |
| N334Q | 7260.045 |  | 9879.347 |  | 0.734871 |
| N345Q | 11042.04 |  | 9231.054 |  | 1.196184 |
| N1780Q | 13661.06 |  | 7751.64 |  | 1.762344 |
|  |  |  |  |  |  |
| **Blot 5** |  |  |  |  |  |
|  |  |  |  |  |  |
| WT | 6789.731 |  | 6711.589 |  | 1.011643 |
| N258Q | 22141.22 |  | 9738.489 |  | 2.273579 |
| N334Q | 13217.59 |  | 10880.196 |  | 1.21483 |
| N345Q | 26022.91 |  | 8986.489 |  | 2.895781 |
| N1780Q | 17076.91 |  | 7250.296 |  | 2.355339 |

**Quantification – Surface**

|  | Cav3.2 |  | NaK ATPase | | Ratio |
| --- | --- | --- | --- | --- | --- |
|  |  |  |  |  |  |
| **Blot 1** |  |  |  |  |  |
| WT | 24480.35 |  | 9363.924 |  | 2.614326 |
| N258Q | 18106.43 |  | 10546.29 |  | 1.716853 |
| N334Q | 9232.61 |  | 13801.07 |  | 0.668978 |
| N345Q | 17354.36 |  | 11296.53 |  | 1.536256 |
| N1780Q | 24872.45 |  | 13617.79 |  | 1.826467 |
|  |  |  |  |  |  |
| **Blot 2** |  |  |  |  |  |
| WT | 17620.64 |  | 8756.56 |  | 2.012279 |
| N258Q | 21441.34 |  | 9808.045 |  | 2.186097 |
| N334Q | 21528.44 |  | 12933.41 |  | 1.66456 |
| N345Q | 5953.882 |  | 10124.51 |  | 0.588066 |
| N1780Q | 2097.669 |  | 11242.75 |  | 0.18658 |
|  |  |  |  |  |  |
| **Blot 3** |  |  |  |  |  |
| WT | 22565.41 |  | 13467.38 |  | 1.675561 |
| N258Q | 26014.31 |  | 19324.57 |  | 1.346178 |
| N334Q | 28487.36 |  | 16152.84 |  | 1.763613 |
| N345Q | 15491.18 |  | 16025.89 |  | 0.966634 |
| N1780Q | 11904.67 |  | 16359.31 |  | 0.7277 |
|  |  |  |  |  |  |
| **Blot 4** |  |  |  |  |  |
| WT | 29851.94 |  | 8202.581 |  | 3.639335 |
| N258Q | 26559.8 |  | 10480.72 |  | 2.534157 |
| N334Q | 26150.85 |  | 7445.631 |  | 3.512241 |
| N345Q | 17375.71 |  | 14521.45 |  | 1.196554 |
| N1780Q | 32507.99 |  | 12723.6 |  | 2.554936 |
|  |  |  |  |  |  |
| **Blot 5** |  |  |  |  |  |
| WT | 20350.06 |  | 28404.35 |  | 0.716442 |
| N258Q | 36288.39 |  | 26537.25 |  | 1.367451 |
| N334Q | 25641.12 |  | 14143.97 |  | 1.812866 |
| N345Q | 4023.255 |  | 20350.79 |  | 0.197695 |
| N1780Q | 9343.894 |  | 23502.3 |  | 0.397574 |
